# Clear cell changes in salivary gland neoplasms: A 20-year retrospective study

**DOI:** 10.4317/medoral.21570

**Published:** 2017-04-08

**Authors:** Tina Woods, Sarah Fitzpatrick, Donald Cohen, Mohammed Islam, Indraneel Bhattacharyya

**Affiliations:** 1 DMD. Department of Oral Maxillofacial Surgery and Pathology, University of Mississippi Medical Center, Jackson, Mississippi, USA; 2 DDS. Department of Oral and Maxillofacial Diagnostic Sciences, University of Florida, Gainesville, Florida, USA; 3 DMD, MS, MBA. Department of Oral and Maxillofacial Diagnostic Sciences, University of Florida, Gainesville, Florida, USA; 4 DDS, BDS. Department of Oral and Maxillofacial Diagnostic Sciences, University of Florida, Gainesville, Florida, USA; 5 DDS, MSD. Department of Oral and Maxillofacial Diagnostic Sciences, University of Florida, Gainesville, Florida, USA

## Abstract

**Background:**

Clear cells are observed histopathologically in both benign and malignant neoplasms but their presence in salivary gland tumors has not been extensively documented.

**Material and Methods:**

With IRB approval, the archive of the University of Florida College of Dentistry oral pathology biopsy service was retrospectively searched from 1994-2014 for all benign and malignant salivary tumors. Epidemiological data, tumor location and duration, and type of tumor were recorded. A four reviewer panel examined the original slides. Reviewers scaled each case as 0 (no clear cells present), 1 (few to focal clear cells), 2 (less than 50% clear cells), and 3 (greater than 50% clear cells).

**Results:**

A total of 535 cases were included of which 48% of tumors displayed 0 clear cells (257/535), 31.4% (168/535) scored 1, 13.6% (73/535) scored 2, and 7% (37/535) scored 3. Of the 251 (47%) malignant neoplasms, 64% (160/251) demonstrated 0-1 clear cell change, while 36% (91/251) showed a score of 2-3. For the total 284 (53%) benign tumors, 93% (265/535) scored 0-1 and 7% (19/535) scored a 2-3 range. No statistical difference was noted for gender, age, or duration of time present in regards to presence or absence of clear cells. Statistically significant differences in clear cell presence were found between location groups, between benign and malignant diagnosis, and between specific diagnostic groups.

**Conclusions:**

This study demonstrates the frequent presence of increased numbers of clear cells in oral salivary malignancies and highlights salivary gland differential diagnoses when presented with clear cell changes.

** Key words:**Clear cell change, salivary tumors, benign tumors, malignant tumors.

## Introduction

Clear cells may be found as incidental histologic findings in a multitude of benign or malignant tumors of many cell origins including epithelial, melanocytic, mesenchymal, or hematopoietic (1). They may be a result of many different processes, including artifact, degeneration of cellular organelles, or accumulation of substances within the cells - most commonly glycogen, but sometimes mucopolysaccharides, mucin, lipids, or foreign bodies (1,2). Within the head and neck region, clear cells are found most commonly in salivary gland tumors, but also may be seen in tumors of squamous or odontogenic epithelial origin, primary or metastatic carcinoma, benign or malignant melanocytic lesions, or benign or malignant mesenchymal tumors (1,3). Knowledge of the frequency and patterns of clear cell presentation in salivary gland tumors may be helpful in determining a diagnosis. This study serves to describe both the epidemiologic, clinical, and histologic features of a large series of salivary tumors and also to detail the occurrence and histologic appearance of clear cells found within this group of tumors.

## Material and Methods

Institutional review board approval and permission for waiver of informed consent was granted from the University of Florida Institutional Review Board Ethics Committee (Approval #IRB201400598). We identified 641 cases of salivary tumors in the archive files of the University of Florida College of Dentistry’s Oral Pathology Laboratory (Gainesville, FL) dating from January 1994- January 2014. Epidemiologic data was collected to include age, gender, diagnosis, site of occurrence, and time period the lesion was present. In instances where both an incisional and excisional specimen were available, the excisional specimen was chosen for examination in the study. Metastatic tumors and cases with insufficient tissue were excluded from the study. Hematoxylin and eosin stained slides were examined for each tumor by a panel of four Oral and Maxillofacial Pathologists (IB, DC, MI, SF). Clear cell changes were tabulated for each case with the following criteria: tumors with no clear cells present = grade 0 (negative); those with few to focal clear cells (less than 25%) = grade 1 (focal); tumors with moderate clear cells over 25% but less than 50% = grade 2 (moderate); tumors composed of greater than 50% clear cells = grade 3 (diffuse). Statistical analysis was performed to compare differences between groups in terms of clear cell composition with Pearson chi-square test using IBM SPSS version 22, and a *p*-value < 0.05 was considered statistically significant.

## Results

**Figure 1:**
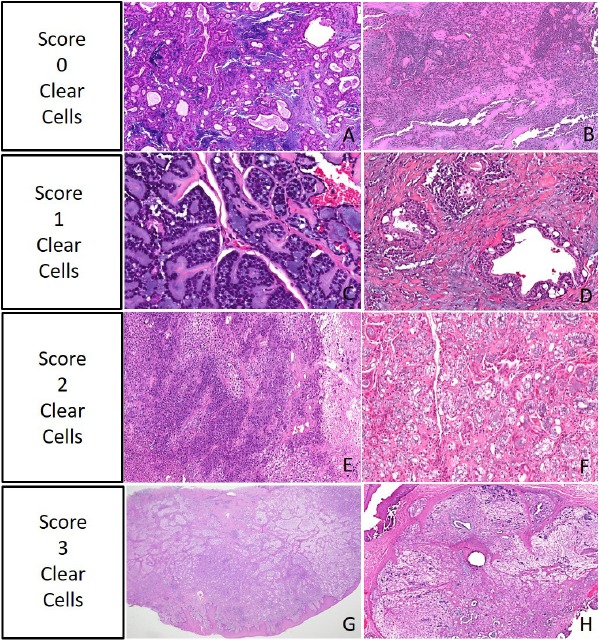
Examples of Grade 0=no clear cells change (A,B), Grade 1=focal ≤25% clear cells change (C,D), Grade 2=moderate 25% -50% clear cells change (E,F), Grade 3=diffuse ≥ 50% clear cells change (G,H). Hematoxylin and eosin magnifi cation 5x (A,B,E,G,H) and 20x (C,D,F).

The final number of cases included in the study was 535. Examples of specimens graded as levels 0-3 may be seen in figure 1. An overall comparison between 0-1 (none to focal) clear cell score to 2-3 (moderate to diffuse) clear cell score in terms of demographics, clinical information, and diagnoses is presented in  Table 1. Of the overall 535 cases analyzed, 425 (79.4%) showed 0-1 grade clear cell change and 110 (20.6%) showed 2-3 grade clear cell change. Gender was documented for all 535 cases, with a distribution of 230 males and 305 females. Age was provided for 517/535 cases showing 171 patients under 50 years of age and the great majority being age 50 and above, with 346. The slight majority of patients reporting duration of symptoms noted the lesions were present less than 1 year at time of biopsy (n=171/335 reported) with the remainder reporting the lesions present over 1 year (n=164/335 reported). There were no statistically significant differences in clear cell group levels in regards to gender, age group, or length of time that symptoms were reported prior to biopsy.

**Table 1:**
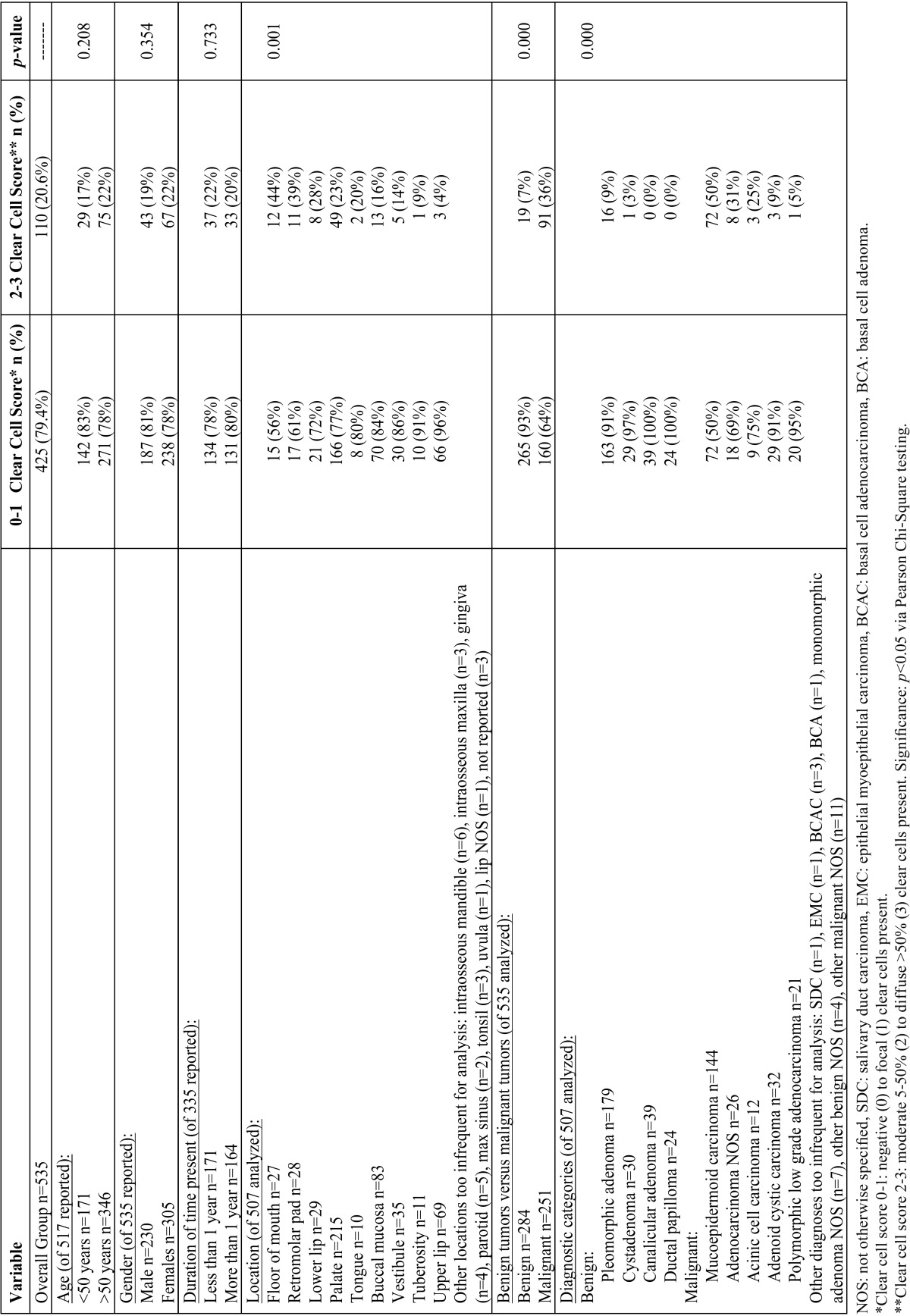
Comparison of clear cell scoring by demographics, clinical data, and diagnosis.

**Figure 2:**
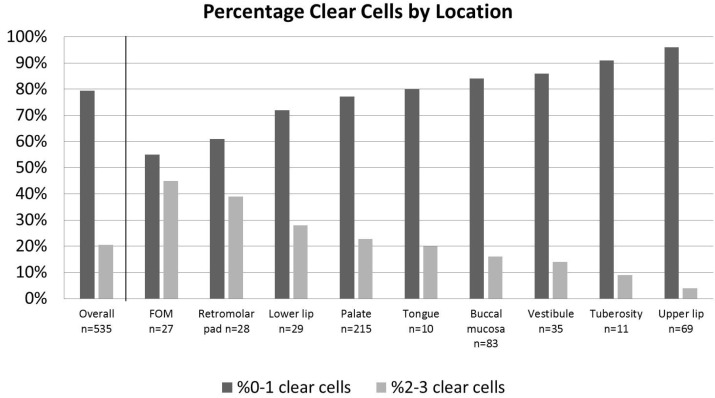
Comparison of clear cell grades 0-1 to grades 2-3 by oral location.

A comparison of clear cell level by oral cavity locations is summarized in figure 2. The majority of clear cell grade 2-3 occurred on the floor of mouth, followed in descending order by the retromolar pad, lower lip, palate, tongue, buccal mucosa, vestibule, maxillary tuberosity and upper lip. A statistically significant difference was noted between locations in terms of clear cell grade 0-1 as compared to grade 2-3 (*p*=0.001).

**Figure 3:**
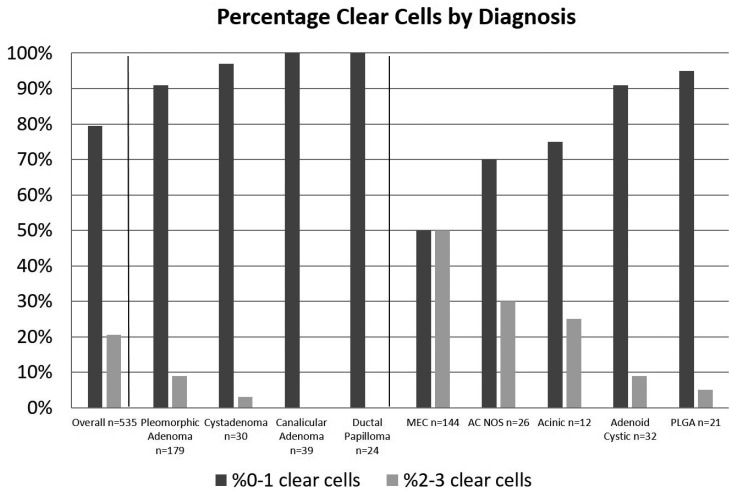
Comparison of clear cell grades 0-1 to grades 2-3 by diagnosis.

Clear cell changes in regards to diagnoses are summarized in figure 3. For benign entities, moderate or diffuse clear cell change was rare, with pleomorphic adenoma (PA) showing the highest rate of 2-3 clear cell change (9%, n=16/179) followed by cystadenoma (including papillary cystadenoma) at 3%, or 1/30 cases. Both the canalicular adenoma (CA) and ductal papilloma groups showed no moderate or diffuse clear cell change in any cases. In malignant tumors, mucoepidermoid carcinoma (MEC) showed the highest rate of clear cell change with an equal 50% grade 0-1 and 50% 2-3 clear cell change. The remaining malignant tumor categories were in descending order of clear cell change adenocarcinoma NOS demonstrating 31% (n=8/26) grade 2-3, and acinic cell adenocarcinoma (AcCC) had 25% (n=3/12) grade 2-3. Adenoid cystic carcinomas (AdCC) had only 9% (n=3/32) with grade 2-3, followed by polymorphous low grade adenocarcinoma (PLGA) with 5% (n=1/21) grade 2-3. Other benign and malignant diagnoses were present only in very small numbers. Between diagnostic categories a statistically significant difference was found between 0-1 clear cell grade and 2-3 clear cell grade (*p*=0.000).

Overall clear cell changes with respect to benign and malignant tumors revealed that out of 284 benign salivary gland tumors, 93% demonstrated clear cell changes with a grade 0-1 while only 7% had grade 2-3 clear cell changes. In contrast, 251 malignant tumors demonstrated 64% grade 0-1 clear cell changes and a higher number of grade 2-3 clear cells of 36%, which was a statistical difference in comparison to benign tumors (*p*=0.000).

## Discussion

The cases described in our series contribute one of the largest case series of salivary gland tumors described in the literature. For the most part, the demographics of the cases in our series are commensurate to the parameters previously reported. The mean age affected was 57.7 years which is similar to overall rates mentioned in the literature for salivary tumors (4). Females comprised 57% of the patients in the study, which is also consistent with most cited reports (4). Due to the nature of our biopsy service which is comprised mainly of submissions from private practice offices not involving a hospital setting, parotid tumor submissions were rare, and most cases represented minor salivary gland based tumors. The benign to malignant ratio in this study was close to equal, which is typical of minor salivary gland tumors (4). PA was the most common benign tumor in the study and MEC the most common malignancy, which fits with prior studies (2,5). As expected, tumors arising mainly in the parotid were infrequent [AcCC, epithelial myoepithelial carcinoma (EMC), salivary duct carcinoma (SDC), basal cell adenoma (BCA), basal cell adeno-carcinoma (BCAC)] or not represented (Warthin tumor, oncocytoma). The most common locations of tumors in our series were palate, followed by buccal mucosa, then upper lip which is consistent with reported locations for minor salivary gland lesions (4).

In considering the likelihood for clear cell change in salivary tumors, it is relevant to review the cellular origin of different types of salivary tumors. Salivary tumors are composed generally of either luminal or ductal epithelial cells, myoepithelial cells, or a mixture of both types (6). Monophasic epithelial cell tumors include AcCC (luminal acinar cells) and salivary duct carcinoma and canalicular adenoma (ductal cells) (6,7). Myoepithelioma and myoepithelial carcinoma represent monophasic myoepithelial tumors (6). Biphasic tumors with both luminal/ductal and myoepithelial elements include PA, EMC, and AdCC (6). The possibility of a role of myoepithelial cells in MEC and PLGA has been questioned but is controversial (8). MEC represents a unique tumor thought to possibly derive from pluripotent reserve cells of the excretory duct which may explain the variation in cell type noted in MEC including squamous, intermediate, and mucous cells (2). In the context of predicting clear cell change, one question arose of which type of salivary gland tumor cell was most likely to exhibit clear cell change. Myoepithelial cells have been observed to rarely show clear cell change due to glycogen accumulation (1,8,9). In addition, the clear cells noted in MEC have been found to be most similar in immunohistochemical profile to the intermediate cells in this tumor (2). In addition, some clear cell predominant salivary tumors are thought to be of squamous origin such as hyalinizing clear cell carcinoma (10). The most common tumors to show clear cell change in this study were tumors with either monophasic or biphasic myoepithelial or luminal origin but not typically solitary ductal origin type tumors.

In the literature, many types of salivary tumors have been described to show clear cell change. Clear cells have been reported in benign tumors such as PA, myoepithelioma, and oncocytoma, and malignant tumors such as myoepithelial carcinoma, oncocytic carcinoma, MEC, AcCC, PLGA, and AdCC (1). Clear cells are commonly associated with MEC, with sporadic clear cell change reported in up to 30% of cases, but predominant clear cell change is rarer (2,11). AcCC has been shown to feature clear cell in approximately 6% of cases, but with predominant clear cell populations in only 1% (1). In another study, light eosinophilic or clear cell change was noted in 35% of AcCC (12). Unlike MEC where clear cells result from glycogen deposition as measured by periodic acid-Schiff (PAS) positivity with loss after diastase, clear cells in AcCC do not show glycogen accumulation and are likely due to a loss of cellular organelles or proliferation of monoclonal clear acinar cells (1). In EMC, the ductal epithelial islands are surrounded commonly by myoepithelial cells exhibiting clear cell change (1,13). Oncocytomas may rarely show clear cell change relating to glycogen deposition or fixation artifact (1,13). Myoepithelial carcinoma may show clear cell change in approximately 16% of cases (1). Clear cell changes have also been reported in AdCC and PLGA, particularly in the solid pattern variants (14). Other salivary tumors are primarily composed of clear cells such as hyalinizing clear cell carcinoma and clear cell carcinoma (10).

Our study found clear cell change to be more common in malignant salivary tumors compared to benign, which is compatible with prior reports (1). To the best of our knowledge, this study is the first to evaluate clear cell change in regards to age, gender, duration of time reported, or location of tumor. We found no significant association of clear cell change with respect to age, gender, or duration of time reported. The association noted with location are explainable primarily due to the predominance of tumors which may occur at those locations, as floor of mouth and retromolar pad had higher than overall clear cell change but had high rate of MEC in these areas (a tumor with high clear cell change), whereas upper lip had a lower than average clear cell rate but a predominance of CA in this location, a tumor which failed to show clear cell change.

We were unable to measure outcome of the tumors due to the retrospective nature of the study and due to the fact that the majority of cases originated from offices outside our institution it was not possible to speculate on clear cells as a predictor of prognosis. Prior studies have found no association between extensive clear cell change and prognosis in either MEC or AcCC (1). However, in other types of tumors clear cell variants have been associated with more aggressive behavior (15).

The differential diagnosis for clear cell predominant tumors of the head and neck is broad and highly dependent on location. Salivary tumors may rarely occur intraosseously and when arising in the jawbones must be distinguished from odontogenic clear cell carcinoma, which may appear very similar to hyalinizing clear cell carcinoma of the salivary gland (10). Salivary lesions proximal to the skin due to tumor extension must be differentiated from a wide range of dermatologic lesions showing clear cell change including epidermal and melanocytic benign and malignant lesions (16). Benign melanocytic clear cell tumors including balloon cell nevus and cellular blue nevus along with malignant melanoma have been reported with predominant clear cell change (16). In addition, multiple benign and malignant adnexal eccrine and sebaceous tumors may show clear cell change (16). Squamous cell carcinoma may also show predominant clear cell populations in the skin in some cases (16). Myoepithelial tumors of soft tissue may show clear cell change in 45-55% of tumors but it is rarely predominant (17). Mesenchymal tumors with reported clear cell change include clear cell sarcoma, benign and malignant smooth muscle tumors, and perivascular epithelioid cell tumors (16). Finally, metastatic tumors such as renal cell carcinoma (RCC) are a significant consideration in evaluating clear cell tumors of the head and neck, but RCC is often distinguishable by a robust vascular network and distinct immunohistochemical profile (1).

## Conclusions

In conclusion, in our study population, tumors of primarily ductal origin rarely showed clear cell change, and malignant tumors were significantly more likely than benign to exhibit more extensive clear cell change. Location was somewhat predictive of clear cell change in this study population, but explainable by prevalence of certain tumors in specific locations. Age, gender, and duration were not predictive of clear cell change. Clinicians and pathologists should carefully consider other clear cell tumors in the differential diagnosis when confronted with a salivary tumor with predominant clear cell change.
